# School climate and learning engagement among secondary vocational students: the chain mediating roles of future self-continuity and academic procrastination

**DOI:** 10.3389/fpsyg.2026.1845521

**Published:** 2026-08-04

**Authors:** Jiasui Xu, Peiqiong Zhao, Mengfei Ye, Qingming Liu

**Affiliations:** 1 School of Physical Education, Shaoxing University, Shaoxing, Zhejiang, China; 2 School of Education and Psychology, Shaoxing University, Shaoxing, Zhejiang, China; 3 Center for Brain, Mind, and Education, Shaoxing University, Shaoxing, Zhejiang, China; 4 Department of Psychiatry, Shaoxing Seventh People’s Hospital (Affiliated Mental Health Center, Medical College of Shaoxing University), Shaoxing, Zhejiang, China

**Keywords:** academic procrastination, future self-continuity, learning engagement, school climate, secondary vocational students

## Abstract

**Background:**

This study aimed to examine the relationship between school climate and learning engagement among secondary vocational students, while also exploring the mediating roles of future self-continuity and academic procrastination.

**Methods:**

A cross-sectional online questionnaire survey was conducted with 620 Grade 10–12 students recruited through convenience sampling from one secondary vocational school in Zhejiang Province, China. Participants completed measures of perceived school climate, future self-continuity, academic procrastination, and learning engagement. Data were analyzed using correlation analyses and a serial mediation model.

**Results:**

The study found a significant positive correlation between school climate and both future self-continuity and learning engagement, while academic procrastination exhibited a negative association with all three variables. School climate was positively associated with learning engagement, not only directly but also through indirect pathways via future self-continuity and academic procrastination, as well as through the serial indirect pathway involving both factors.

**Conclusion:**

These findings may provide valuable insights into potential pathways linking school climate and learning engagement and offer implications for supporting learning engagement among secondary vocational students in this sample.

## 1 Introduction

Secondary vocational students constitute an important group in China’s upper-secondary education system, and their learning engagement deserves focused scholarly attention. In this population, learning engagement may need to be understood in relation to the school climate, future self-continuity, and academic procrastination. Against this background, the present study examined the association between school climate and learning engagement and tested the statistical mediating roles of future self-continuity and academic procrastination among Chinese secondary vocational students.

### 1.1 Secondary vocational education context and relevance of the study variables

Technical and vocational education and training broadly refers to education, training, and skills development related to occupational fields, production, services, and livelihoods (UNESCO, 2015). In China, secondary vocational education represents an important upper-secondary educational pathway, with 12.29 million students enrolled nationwide in 2024 (Ministry of Education of the People’s Republic of China, 2024). Accordingly, students in this pathway are expected to engage in school-based academic learning while also connecting coursework and practical training with future occupational roles.

Secondary vocational students may also face distinctive academic and psychosocial challenges. Some students enter secondary vocational schools after academic selection and may have experienced earlier academic frustration (Liu et al., 2022). In addition, vocational education may carry social stigma in some contexts, including stereotypes such as “bad students go to vocational schools” (Ling, 2015). These experiences may be associated with students’ school identity, learning confidence, and future expectations.

These contextual features suggest that school climate, future self-continuity, and academic procrastination are particularly relevant to learning engagement. A supportive school climate may provide important contextual resources, as school cooperation climate has been linked to school belonging and academic performance among Chinese senior-secondary vocational students (Liu et al., 2022). Future self-continuity is relevant because vocational students need to connect present learning with future career development (Chishima and Wilson, 2021). Academic procrastination is also relevant because it reflects whether students can translate learning intentions into timely academic action, a concern examined among Chinese secondary vocational students (Li and Sun, 2023). Taken together, these considerations provide the contextual rationale for examining these variables among secondary vocational students.

### 1.2 School climate and learning engagement

Learning engagement is a multidimensional construct that includes behavioral, emotional, and cognitive components (Fredricks et al., 2004). Given academic deadlines and task demands, learning engagement is a key variable for understanding students’ learning processes (Yousefi Afrashteh and Janjani, 2024). In the present study, school climate refers to students’ overall perceptions of the school learning environment (Gilman et al., 2021), primarily reflected in teacher support, peer support, and autonomous opportunity. Prior studies have shown that school climate is closely related to students’ learning motivation (Fan and Williams, 2018), learning engagement (Lombardi et al., 2019), and academic achievement (Berkowitz et al., 2017). However, empirical evidence regarding the association between school climate and learning engagement among Chinese secondary vocational students remains limited. Accordingly, the present study first examined whether school climate was positively associated with learning engagement in this population.

Conservation of Resources (COR) theory, introduced by Stevan E. Hobfoll, underscores the pivotal role of “resources” in shaping individuals’ psychological and behavioral processes (Hobfoll, 1989). According to this theory, individuals with more resources are more likely to acquire additional resources, and such resource availability has been associated with lower stress and more positive psychological and behavioral outcomes (Halbesleben and Wheeler, 2008). Within this theoretical framework, school climate can be conceptualized as a contextual resource, forming part of the “resource caravan passageways” (Hobfoll, 2011). A supportive school climate can help students preserve psychological resources under academic stress and may be associated with access to additional resources, such as respect, care, and assistance, which may support students’ engagement in learning (Hobfoll, 1989; Chen, 2025).

A substantial body of empirical research has substantiated the association between school climate and student engagement in learning. For instance, Cornell et al. (2016) demonstrated that supportive teacher-student relationships and the perception of fairness in school discipline and regulations are linked to elevated levels of academic engagement. Similarly, a longitudinal study conducted by Del Toro and Wang (2021) indicated that students’ perceptions of a positive school climate are correlated with increased academic engagement over time. In environments characterized by emotional support and care, students are more willing to express their ideas openly and exhibit a more positive attitude toward academic pursuits (Wang and Eccles, 2013; Li et al., 2023). Furthermore, a positive school climate has been associated with stronger emotional connections between students and their school, better behavioral regulation, pursuit of academically valued goals, and active participation in learning activities (Yu et al., 2023). Taken together, prior studies suggest that school climate is positively associated with learning engagement, but much of this evidence has been derived from general student populations. Less attention has been paid to this association in Chinese secondary vocational education, where school climate may function as an important contextual resource for students’ learning engagement. Based on COR theory and prior empirical findings, we expected school climate to be positively associated with learning engagement among secondary vocational students.

### 1.3 The mediating role of future self-continuity

The future self refers to an individual’s mental representation of who they may become at a later point in life, including anticipated, hoped-for, or feared versions of the self (Markus and Nurius, 1986). Such representations are closely related to identity because individuals can project themselves into personally meaningful future events (D’Argembeau et al., 2012). Building on this concept, future self-continuity (FSC) refers to the perceived psychological connection between one’s present self and future self, or the extent to which individuals experience their future self as connected to and continuous with their present self (Ersner-Hershfield et al., 2009a; Hershfield, 2011; Sokol and Serper, 2019).

Future self-continuity may be particularly relevant for secondary vocational students because they need to connect current academic learning and practical training with future occupational development. Possible selves theory suggests that how individuals imagine who they may become is linked to identity, motivation, and goal-directed behavior (Markus and Nurius, 1986), while future self-continuity is closely related to intertemporal decision making and future-oriented behavior (Hershfield, 2011). Empirically, future self-continuity has been found to be associated with academic and career planning and to be lower among vocational-oriented high school students than among academically oriented students (Chishima and Wilson, 2021). Taken together, previous research suggests that future self-continuity is relevant to future-oriented decision-making, academic and career planning, and learning-related outcomes. However, less attention has been paid to how future self-continuity may be associated with school climate among secondary vocational students. Therefore, future self-continuity was examined as a psychological mediator in the association between school climate and learning engagement because it reflects students’ perceived connection between their present and future selves in vocational education contexts.

From an ecological perspective, school climate may be an important contextual correlate of students’ future self-continuity. School is one of the most immediate microsystems related to students’ development, in addition to the family environment (Bronfenbrenner and Evans, 2000; Eccles and Roeser, 2011). Efforts to improve school climate are rooted in the bio-ecological theory of child and adolescent development, which emphasizes that individual, family, school, and broader environmental characteristics are associated with learning and behavior (Thapa et al., 2013). Within educational contexts, teachers and peers constitute primary sources of social support. Empirical research has indicated that support from peers and teachers is associated with students’ hope (Xiang et al., 2020). Individuals with elevated levels of hope are inclined to possess more positive and vivid expectations regarding the future. Positive anticipation of one’s future self is a crucial aspect of future self-continuity (Ersner-Hershfield et al., 2009a; Ersner-Hershfield et al., 2009b), with positivity and vividness being fundamental dimensions of this continuity (Hershfield, 2011). Previous studies have demonstrated that when students possess an unclear or incoherent vision of their future selves—characterized by low future self-continuity—supportive relationships with peers and teachers, along with a positive school climate, may serve as alternative sources of meaning, reward, and hope (Wei et al., 2026). Furthermore, the external structure and guidance inherent in the school environment may help compensate for weak internalization of the future self (Meng et al., 2024).

The manner in which individuals connect their future self with their present self is associated with the concept of “foresight” (Bartels and Urminsky, 2011). According to Hershfield’s theoretical model of future self-continuity, individuals with higher levels of future self-continuity are more motivated to care for their future self and may evaluate it more positively. These individuals also demonstrate future-oriented decisions and behaviors (Ersner-Hershfield et al., 2009b; Urminsky, 2017). Learners with higher future self-continuity may be more likely to envision positive future scenarios, which has been linked to higher learning engagement and better academic performance. This orientation may encourage them to “save” and “invest” in their future selves (Yang et al., 2024). Taken together, a supportive school climate may be associated with higher future self-continuity among secondary vocational students, which may provide a future-oriented motivational basis for their learning engagement.

### 1.4 The mediating role of academic procrastination

Academic procrastination refers to the intentional postponement of course-related tasks despite awareness of possible adverse outcomes (Ye et al., 2025). It is commonly regarded as a form of irrational delay (Yan and Zhang, 2022) and has been linked to diminished motivation for goal pursuit, difficulties in self-regulation, poorer academic performance, lower emotional well-being, and lower life satisfaction (Steel, 2007; Krause and Freund, 2016; Stead et al., 2010; Kim and Seo, 2015; Yue et al., 2024).

Academic procrastination is relevant to the present model because it represents a proximal behavioral manifestation of self-regulatory difficulty in learning. Compared with broader motivational constructs, it more directly reflects whether students translate learning intentions into timely academic action. Temporal motivation theory suggests that procrastination is related to expectancy, task value, impulsiveness, and delay (Steel and König, 2006), and it has also been conceptualized as a form of self-regulatory failure and short-term mood regulation (Steel, 2007; Sirois and Pychyl, 2013). Therefore, academic procrastination may capture a learning-related behavioral pathway linking school climate, future self-continuity, and learning engagement. In addition, research among Chinese secondary vocational students has shown that future-oriented psychological resources, such as future time insight, are associated with academic procrastination and self-control (Li and Sun, 2023).

Existing studies also suggest that academic procrastination is related to both contextual factors and learning engagement. Parenting practices, the school environment, and individual factors have been found to be associated with academic procrastination (Zheng et al., 2023; Kamber et al., 2024). Within a positive school environment, students’ emotional interactions with teachers and peers may be linked to a lower likelihood of procrastination (Touloupis and Campbell, 2024). Conversely, students situated in negative environments may experience fewer supportive or protective factors and may be more likely to use academic procrastination as an avoidant response to immediate academic stress (Yue et al., 2021). Academic procrastination has also been associated with diminished enthusiasm and interest in learning, lower learning engagement, superficial effort, lower learning efficiency, and inattentiveness in class (Li et al., 2023).

Taken together, existing studies indicate that academic procrastination is closely related to self-regulation, contextual support, and learning-related outcomes. However, less attention has been paid to how academic procrastination may operate together with future self-continuity in the association between school climate and learning engagement. Therefore, the present study examined academic procrastination as a mediator in this association, both independently and in sequence with future self-continuity.

### 1.5 The chain mediating role of future self-continuity and academic procrastination

Future self-continuity and academic procrastination may form a sequential pathway linking school climate and learning engagement. Future self-continuity concerns the perceived psychological connection between the present and future self and is closely related to intertemporal decision-making and future-oriented behavior (Hershfield, 2011; Urminsky, 2017). Procrastination has been conceptualized as a form of self-regulatory failure (Steel, 2007) and as a short-term mood-regulation strategy whose negative consequences are often borne by the future self (Sirois and Pychyl, 2013). Empirical research has also shown that future self-continuity is associated with academic procrastination (Blouin-Hudon and Pychyl, 2015). Thus, stronger future self-continuity may be associated with lower academic procrastination, which may in turn support greater learning engagement by helping students sustain effort and participation in learning activities.

**Figure 1:**
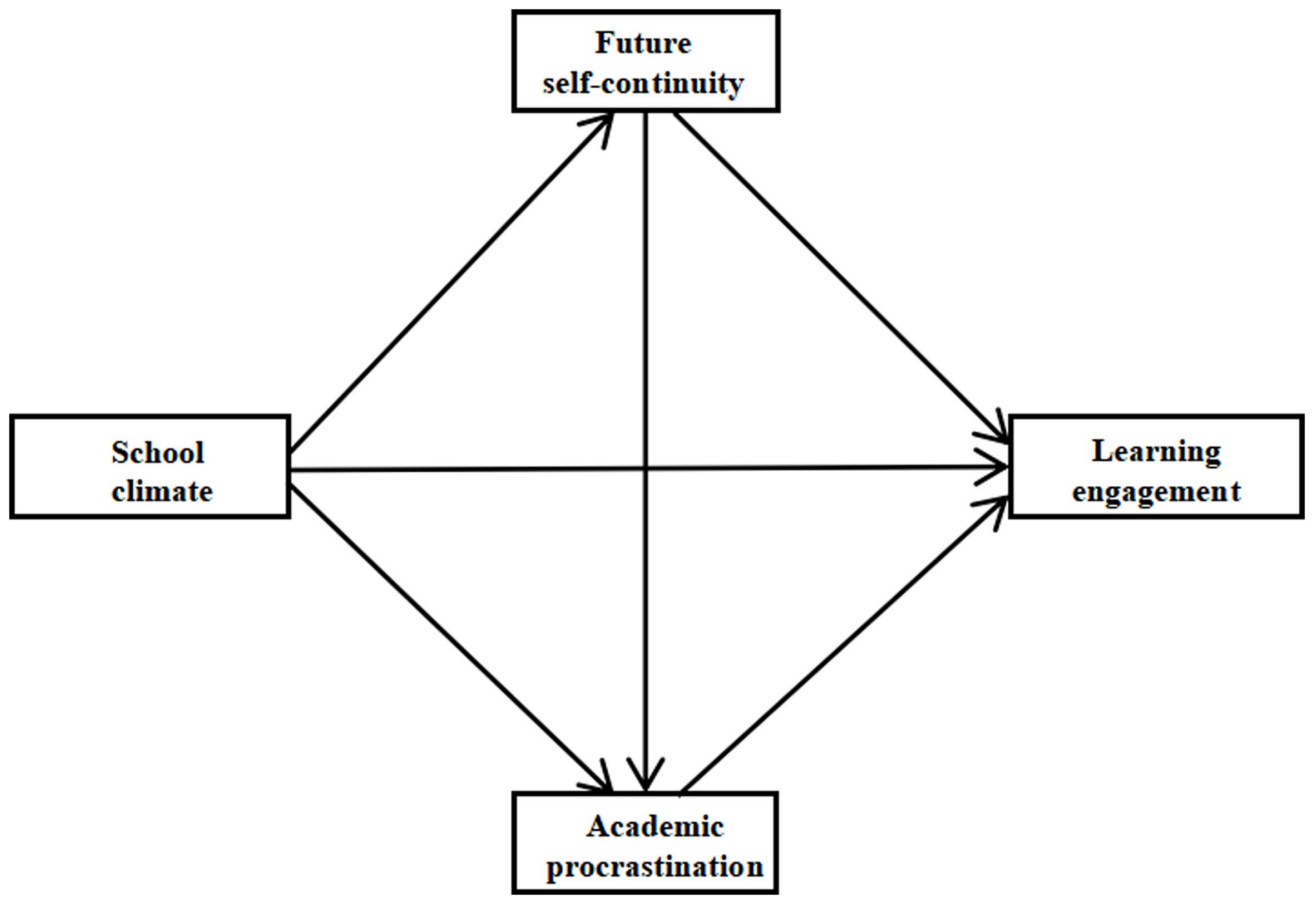
Diagram of the theoretical hypothetical model.

This study developed a chain mediation model (see Figure 1), utilizing survey data from secondary vocational students in China to investigate the relationship between school climate and learning engagement, alongside examining the statistical mediating roles of future self-continuity and academic procrastination. The present study addresses two related gaps. First, although school climate and learning engagement have been widely examined in general student populations, their association remains less clearly understood among Chinese secondary vocational students, who face distinctive academic and psychosocial challenges. Second, although future self-continuity and academic procrastination have each been examined in relation to students’ learning-related outcomes, their combined and sequential roles in the association between school climate and learning engagement remain underexamined, particularly among Chinese secondary vocational students. By integrating school climate, future self-continuity, and academic procrastination into a serial mediation model, this study contributes to the literature by extending learning engagement research to Chinese secondary vocational students and by providing preliminary evidence for potential statistical indirect pathways involving future self-continuity and academic procrastination. The hypotheses proposed were as follows:

*H1*: There is a significant positive association between school climate and learning engagement among secondary vocational students.

*H2*: Future self-continuity serves as a mediator in the relationship between school climate and learning engagement in this student population.

*H3*: Academic procrastination functions as a mediator in the relationship between school climate and learning engagement among secondary vocational students.

*H4*: School climate is expected to be indirectly associated with learning engagement through the serial pathway involving future self-continuity and academic procrastination.

## 2 Methods

### 2.1 Participants

A convenience sampling approach was used to collect data through an online survey platform from students at a secondary vocational school in Zhejiang Province, China. After permission was obtained from school authorities, the survey link was shared with students. Participation was voluntary, informed consent was obtained, and responses were kept confidential and anonymous. The study was approved by the local ethics committee. Of the 678 questionnaires distributed, 620 valid responses were obtained, resulting in a valid response rate of 91.45%.

The final analytic sample was mainly drawn from participating classes in several vocational program areas, with a large proportion of participants coming from Preschool Education, Nursing, and Applied Chemistry. The sample comprised 80 male students (12.90%) and 540 female students (87.10%). Because the participating classes were concentrated mainly in program areas that tend to enroll a higher proportion of female students, particularly Preschool Education and Nursing, female students accounted for a larger proportion of the final analytic sample. No gender-based inclusion or exclusion criteria were used. Participants were enrolled in Grades 10–12, including 201 Grade 10 students, 283 Grade 11 students, and 136 Grade 12 students. These grade levels indicate that the sample represented students at the upper-secondary vocational education stage. Age was not included as an item in the original questionnaire. Therefore, exact age-related descriptive statistics, including the mean, standard deviation, and range, were not available.

### 2.2 Measures

All instruments used in this study were established scales with prior validation evidence in relevant student or adolescent samples. The item content, dimensional structure, and scoring procedures were not modified, and internal consistency was further examined in the present sample using Cronbach’s *α*.

#### 2.2.1 School climate scale

The Perceived School Climate Scale developed by Jia et al. (2009) was used to assess students’ perceptions of school climate. The scale contains 25 items across three dimensions: teacher support, peer support, and autonomy opportunities. This scale has demonstrated satisfactory psychometric properties in adolescent samples, including Chinese adolescents (Jia et al., 2009), and has subsequently been applied with acceptable reliability in Chinese educational-context studies involving college students and left-behind adolescents (Wang et al., 2017; Zhang R. P. et al., 2022). Items are rated on a 4-point Likert scale ranging from 1 (never) to 4 (always), with seven items reverse scored. Higher scores indicate a more positive perceived school climate. An example item is: “Teachers help students solve problems at school.” In the present study, the scale demonstrated high internal consistency, with Cronbach’s *α* = 0.934, and dimension-specific reliabilities of 0.891, 0.897, and 0.807. Confirmatory factor analysis yielded *χ^2^* = 833.43, df = 272, RMSEA = 0.06, CFI = 0.92, GFI = 0.91, TLI = 0.90.

#### 2.2.2 Future self-continuity scale

The Chinese version of the Future Self-Continuity Questionnaire, adapted by Zhang F. et al. (2022) from Sokol and Serper’s (2020) original scale, was used to assess students’ perceived psychological connection between their present and future selves. The questionnaire consists of 10 items across three dimensions: similarity, vividness, and positivity. The Chinese version has shown satisfactory reliability and validity in prior validation research (Zhang F. et al., 2022; Zhang R. P. et al., 2022) and has subsequently been applied with acceptable reliability in studies involving college students and academic procrastination-related research (Wang S. et al., 2025; Song et al., 2025). Items are rated on a 6-point Likert scale, with higher scores indicating greater future self-continuity. An example item is: “How similar will your thoughts now be to your thoughts 10 years from now?” In the present study, the questionnaire demonstrated good internal consistency, with Cronbach’s *α* = 0.849 overall, and dimension-specific reliabilities of 0.883, 0.760, and 0.943. Confirmatory factor analysis yielded *χ^2^* = 59.60, df = 32, RMSEA = 0.04, CFI = 0.99, GFI = 0.99, TLI = 0.99.

#### 2.2.3 Academic procrastination scale

Academic procrastination was measured using the Aitken Procrastination Inventory (Aitken, 1982), revised by Liu and Lu (2011). The Chinese revision has demonstrated acceptable reliability and validity in Chinese secondary school student samples (Liu and Lu, 2011) and has also been used in previous studies with good psychometric evidence (Yue et al., 2021; Chen Y. et al., 2024; Wang Y. Y. et al., 2025). This 13-item tool assesses task aversion and fear of failure, with responses on a 5-point Likert scale. Six items are reverse scored. An example statement is: “I always wait until a learning task can no longer be postponed before I begin to work on it.” Higher total scores indicate more severe procrastination. In the present study, the scale showed good internal consistency, with Cronbach’s *α* = 0.878. Confirmatory factor analysis yielded *χ^2^* = 176.79, df = 64, RMSEA = 0.06, CFI = 0.90, GFI = 0.97, TLI = 0.88.

#### 2.2.4 Learning engagement scale

Learning engagement was measured using the Chinese version of the Utrecht Work Engagement Scale-Student, originally developed by Schaufeli et al. (2002a) and Schaufeli et al. (2002b) and translated into Chinese by Fang et al. (2008). The Chinese version has been validated in Chinese student samples and has shown satisfactory reliability and validity (Fang et al., 2008). It has also been applied with good psychometric evidence in subsequent Chinese high school student samples (Jia et al., 2020; Wu and Guo, 2024). The scale includes 17 items across three dimensions: vigor (6 items), dedication (5 items), and absorption (6 items). Items are rated on a 7-point Likert scale, ranging from 1 (never) to 7 (always/every day). An example item is: “Even when my studies do not go well, I am not discouraged and can persist.” Higher total scores indicate higher levels of learning engagement. In the present study, the internal consistency was good, with Cronbach’s α = 0.935 for vigor, 0.943 for dedication, 0.944 for absorption, and 0.974 for the total scale. Confirmatory factor analysis yielded *χ^2^* = 482.72, df = 116, RMSEA = 0.07, CFI = 0.94, GFI = 0.95, TLI = 0.91.

### 2.3 Statistical analysis

Descriptive statistics, correlation analyses, and chain mediation model tests were performed using SPSS 27.0 and PROCESS v4.0. Potential common method bias was preliminarily assessed using Harman’s single-factor test. All measurement items were entered into an unrotated exploratory factor analysis. Given the limitations of this approach, the result was interpreted as preliminary evidence rather than as a definitive test ruling out common method bias. Pearson correlations assessed links among school climate, future self-continuity, academic procrastination, and learning engagement, while Spearman correlations examined demographic variables’ associations with study variables. In the chain mediation model, school climate was the independent variable, learning engagement the dependent variable, and future self-continuity and academic procrastination were mediators. Gender, grade level, urban–rural origin, class leadership experience, father’s educational attainment, and mother’s educational attainment were included as control variables in the serial mediation analyses. These variables were selected because preliminary Spearman correlation analyses showed that they were differentially associated with the study variables. All variables were standardized, and PROCESS v4.0 Model 6 was used to estimate the serial mediation model, with all covariates entered simultaneously in each regression equation. The significance of indirect effects was assessed using the Bootstrap method with 5,000 samples, considering an effect significant if the 95% confidence interval excluded zero.

## 3 Results

### 3.1 Common method bias analysis

Harman’s one-factor test was used to check for common method bias through exploratory factor analysis of all variables. The first factor explained 36.56% of the variance, which was below the 40% threshold. This result suggests that a single dominant common factor was not evident in the data. Nevertheless, because all main variables were collected from the same respondents at the same time using self-report questionnaires, the possibility of common method bias should still be acknowledged when interpreting the findings.

### 3.2 Descriptive statistics and correlation analysis

The socio-demographic characteristics of the participants are detailed in Table 1. Table 2 provides the means and standard deviations for the variables of school climate, future self-continuity, academic procrastination, and learning engagement. Pearson correlation analyses revealed that school climate is significantly and positively associated with future self-continuity and learning engagement (*r* = 0.364–0.637, *p* < 0.01). Conversely, academic procrastination is significantly and negatively associated with school climate, future self-continuity, and learning engagement (*r* = −0.689 to −0.288, *p* < 0.01). Furthermore, Spearman correlation analyses indicated that grade level, gender, place of origin, class leadership experience, and parents’ educational level exhibit differential relationships with the study variables. Consequently, these variables were incorporated as covariates in the chain mediation analyses. Additional current-sample evidence for measurement validity was also examined. Standardized factor loadings were summarized in Table 2 and ranged from 0.701 to 0.859 across the four instruments. Overall, the composite reliability (CR) values ranged from 0.774 to 0.830, and the average variance extracted (AVE) values ranged from 0.590 to 0.632, meeting commonly used criteria for convergent validity (Fornell and Larcker, 1981). In addition, the square roots of the AVE values were greater than the corresponding inter-construct correlations, supporting discriminant validity according to the Fornell-Larcker criterion. Together with the CFA results, these analyses provided additional current-sample evidence for the psychometric adequacy of the adopted instruments.

**Table 1:** Socio-demographic characteristics (*n* = 620).

Variables	*n*	%
Gender		
Male	80	12.90
Female	540	87.10
Grade		
Grade 10 (Year 1)	201	32.42
Grade 11 (Year 2)	283	45.65
Grade 12 (Year 3)	136	21.94
Urban–rural origin		
Urban	336	54.19
Rural	284	45.81
Class leadership experience		
Yes	322	51.94
No	298	48.06
Father’s educational attainment		
No formal education	1	0.16
Primary school	19	3.06
Junior middle school	183	29.52
Secondary vocational school	99	15.97
General senior high school	117	18.87
Junior college	81	13.06
Bachelor’s degree	109	17.58
Postgraduate degree or above	11	1.77
Mother’s educational attainment		
No formal education	1	0.16
Primary school	47	7.58
Junior middle school	179	28.87
Secondary vocational school	93	15.00
General senior high school	86	13.87
Junior college	85	13.71
Bachelor’s degree	116	18.71
Postgraduate degree or above	13	2.10

**Table 2:** The results of correlation analysis between variables (*n* = 620).

Variables		M	SD	FL	CR	AVE	1	2	3	4	5	6	7	8	9	10
1	Gender	–	–	–	–	–	–									
2	Grade level	–	–	–	–	–	0.105**	–								
3	Urban–rural origin	–	–	–	–	–	0.035	0.167**	–							
4	Class leadership experience	–	–	–	–	–	0.014	0.082*	0.036	–						
5	Father’s educational attainment	–	–	–	–	–	−0.044	−0.102*	−0.363**	−0.157**	–					
6	Mother’s educational attainment	–	–	–	–	–	−0.064	−0.125**	−0.337**	−0.129**	0.602**	–				
7	School climate	3.18	0.46	0.722–0.832	0.830	0.621	0.041	−0.151**	−0.074	−0.106**	0.056	0.084*	**0.788**			
8	Future self-continuity	3.89	0.72	0.739–0.790	0.812	0.590	−0.003	−0.061	−0.058	−0.128**	0.111**	0.109**	0.364**	**0.768**		
9	Academic procrastination	2.30	0.58	0.733–0.853	0.774	0.632	−0.119**	0.132**	0.088*	0.099*	−0.114**	−0.096*	−0.568**	−0.288**	**0.795**	
10	Learning engagement	4.64	1.22	0.701–0.859	0.813	0.594	0.036	−0.125**	−0.108**	−0.121**	0.184**	0.132**	0.637**	0.382**	−0.689**	**0.771**

M, Mean; SD, standard deviation; FL, factor loadings; CR, composite reliability; AVE, average variance extracted. Values in bold on the diagonal for the four study variables represent the square roots of AVE. *p < 0.05, **p < 0.01, ***p < 0.001.

### 3.3 Mediation effects test

**Figure 2:**
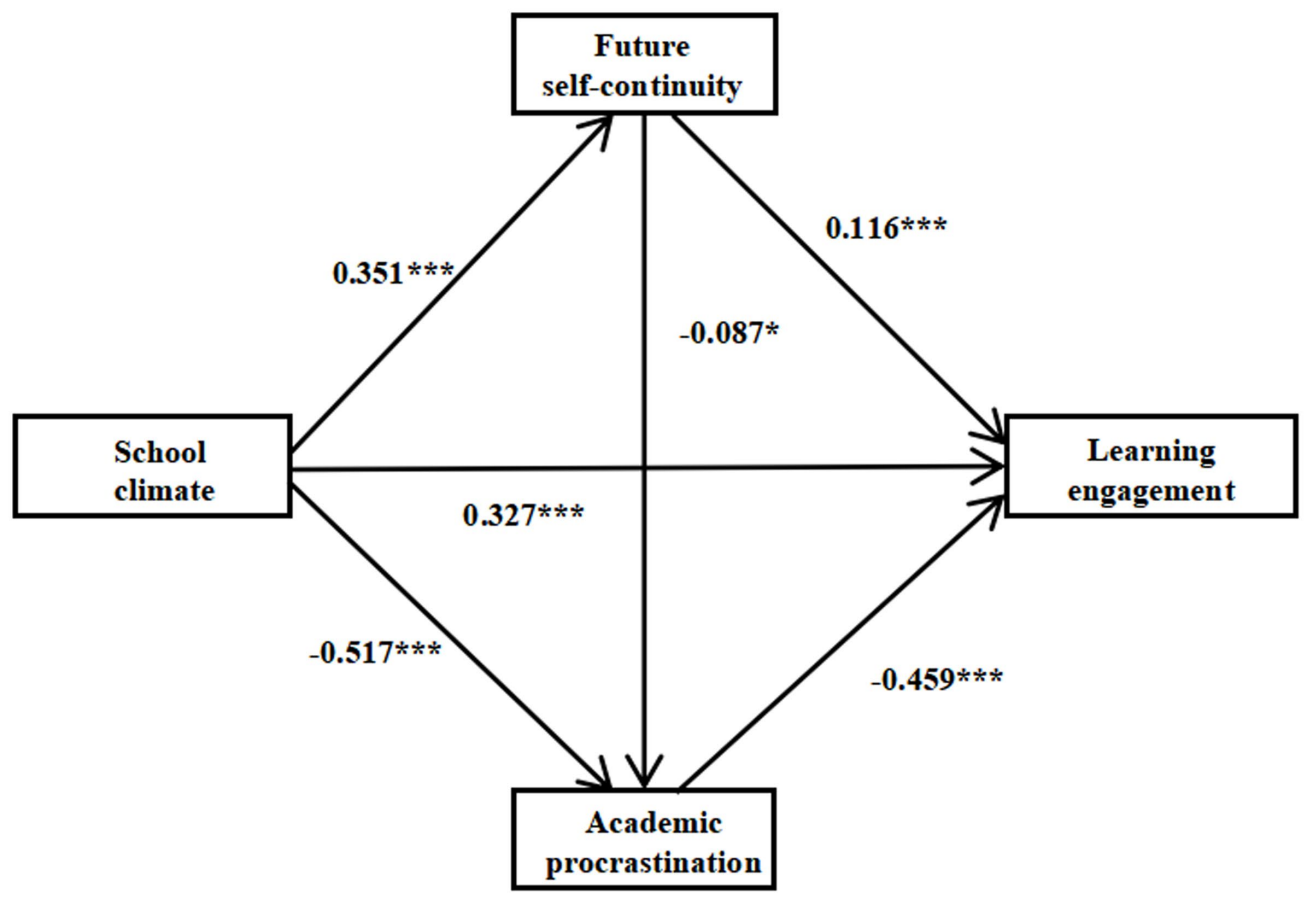
Diagram of the chain mediating effect model. *p < 0.05, **p < 0.01, ***p < 0.001.

The variables of school climate, future self-continuity, academic procrastination, and learning engagement were standardized for analysis. A chain mediation model (Model 6) was evaluated using the PROCESS. The findings, as presented in Table 3 and Figure 2, indicated that the overall association between school climate and learning engagement, in the absence of mediators, was significant (*β* = 0.619, *t* = 19.885, *p* < 0.001). The coefficients and t-values of all covariates, together with the main regression paths in the serial mediation model, are reported in Table 3. Upon incorporating future self-continuity and academic procrastination into the mediation model, school climate was significantly associated with future self-continuity (*β* = 0.351, *t* = 9.231, *p* < 0.001), academic procrastination (*β* = −0.517, *t* = −14.560, *p* < 0.001), and learning engagement (*β* = 0.327, *t* = 9.946, *p* < 0.001). Furthermore, future self-continuity was significantly associated with both academic procrastination (*β* = −0.087, *t* = −2.461, *p* < 0.05) and learning engagement (*β* = 0.116, *t* = 4.103, *p* < 0.001). Additionally, academic procrastination was significantly associated with learning engagement (*β* = −0.459, *t* = −14.253, *p* < 0.001). Regarding explanatory power, the model predicting learning engagement from school climate and covariates before adding mediators explained 42.9% of the variance. In the serial mediation model, the model predicting future self-continuity explained 14.9% of the variance, the model predicting academic procrastination explained 34.8%, and the full model predicting learning engagement explained 58.7%.

**Table 3:** The test results of mediating effects (*n* = 620).

Predictor variable	Learning engagement		Future self-continuity		Academic procrastination		Learning engagement	
	*β*	*t*	*β*	*t*	*β*	*t*	*β*	*t*
Gender	0.031	0.997	−0.019	−0.514	−0.101	−3.074**	−0.013	−0.484
Grade level	−0.024	−0.768	0.002	0.053	0.053	1.556	0.000	−0.016
Urban–rural origin	−0.002	−0.064	0.012	0.295	0.022	0.615	0.006	0.213
Class leadership experience	−0.046	−1.463	−0.069	−1.806	0.025	0.742	−0.024	−0.882
Father’s educational attainment	0.128	3.218	0.064	1.327	−0.050	−1.169	0.095	2.799
Mother’s educational attainment	0.008	0.208	0.048	1.003	−0.004	−0.087	−0.001	−0.031
School climate	0.619	19.885***	0.351	9.232***	−0.517	−14.560***	0.327	9.946***
Future self-continuity					−0.087	−2.461*	0.116	4.103***
Academic procrastination							−0.459	−14.253***
*R* ^2^	0.429		0.149		0.348		0.587	
*F*	65.667***		15.303***		40.670***		96.410***	

*p < 0.05, **p < 0.01, ***p < 0.001.

To assess the significance of the indirect pathways between school climate and learning engagement, a bootstrapping method with 5,000 resamples was employed to generate 95% confidence intervals. An indirect pathway was deemed significant if the 95% confidence interval excluded zero. The findings, as presented in Table 4, indicated three significant indirect pathways between school climate and learning engagement. The total effect was 0.619. The direct effect was 0.327, accounting for 52.77% of the total effect, whereas the total indirect effect was 0.292, accounting for 47.23% of the total effect. With respect to the relative magnitude of the indirect pathways, the pathway through academic procrastination accounted for the largest proportion of the total effect (38.37%), whereas the pathway through future self-continuity and the serial pathway accounted for smaller but statistically significant proportions of the total effect (6.59 and 2.26%, respectively). This pattern suggests that the pathway through academic procrastination represented the largest statistical indirect component in the model, while future self-continuity contributed to the association both independently and sequentially.

**Table 4:** Results of the Bootstrap method of the mediating effect test.

Effect pathway	Efficiency value	SE	95% Confidence interval		Relative mediation effects
			Lower limit	Upper limit	
Total effect	0.619	0.031	0.558	0.680	
Direct effect	0.327	0.033	0.262	0.391	52.77%
Indirect effect	0.292	0.026	0.244	0.344	47.23%
School climate → Future self-continuity → Learning engagement	0.041	0.012	0.020	0.066	6.59%
School climate → Academic procrastination →Learning engagement	0.238	0.025	0.190	0.289	38.37%
School climate → Future self-continuity → Academic procrastination → Learning engagement	0.014	0.007	0.002	0.028	2.26%

## 4 Discussion

Previous research suggests that while some secondary vocational students may have experienced earlier academic difficulties, they may also show learning potential and motivation in vocational settings (Tan et al., 2022). The present findings should therefore be interpreted in light of the secondary vocational education context, in which students are expected to connect school-based learning with future occupational roles. The current study examined the relationship between school climate and learning engagement among secondary vocational students and tested the mediating roles of future self-continuity and academic procrastination. The findings showed that school climate was positively associated with learning engagement and that this association was statistically mediated by future self-continuity and academic procrastination.

### 4.1 The relationship between school climate and learning engagement

The findings indicate that school climate, as a significant environmental factor, was positively associated with learning engagement, thereby corroborating H1. This result is consistent with prior research (Chen S. et al., 2024; Chen, 2025) and may be understood within the conservation of resources theory, in which school climate can be conceptualized as a contextual resource. A favorable school climate is often characterized by a sense of belonging, supportive interpersonal relationships, and opportunities for autonomous expression and decision-making (Gao et al., 2024). Emotional and academic support from teachers encourages students to actively participate in classroom activities and to invest greater effort in their academic pursuits (Zou et al., 2007; Gao et al., 2023). Moreover, a supportive school climate may facilitate the development of positive peer relationships among students, which are linked to increased levels of learning engagement. This is evidenced by active participation in class, a greater initiative in completing assignments, and a proactive pursuit of additional learning opportunities (Wentzel et al., 2021). Positive perceptions of these aspects of the school climate may enable students to identify with the school community, fostering positive emotions and behaviors aligned with the school’s prevailing norms and values. According to self-determination theory (Ryan and Deci, 2020), school environments that meet students’ basic psychological needs may be related to stronger intrinsic motivation and greater engagement in academic tasks. (Schenkenfelder et al., 2020). In the secondary vocational education context, this finding may be understood in relation to students’ need for supportive school experiences. Because students in this pathway are expected to connect school-based learning with future occupational roles, teacher support, peer support, and autonomy opportunities may help them view school learning as meaningful and remain engaged.

### 4.2 The mediating role of future self-continuity

The study identified that future self-continuity mediated the association between school climate and learning engagement, thereby corroborating H2. A positive school climate is characterized by increased teacher support, peer support, and opportunities for autonomy for students. According to self-determination theory and stage–environment fit theory, students may show more adaptive development when the school environment is aligned with their needs for competence, autonomy, and relatedness (Deci and Ryan, 2000; Eccles et al., 1993; Wang and Eccles, 2013). In a nurturing and supportive school setting, students’ needs for relatedness are more likely to be fulfilled (Wang and Eccles, 2013). Under such conditions, students are likely to exhibit enhanced self-regulation and healthier psychological development. Additionally, they may be more inclined to perceive their “future self” as a continuation of their present self.

Wu et al. (2023) identified that social support was associated with more optimistic future-oriented thinking. Within a supportive school environment, students may report more positive self-assessments and establish future objectives, which correlate with elevated levels of future self-continuity. These findings align with prior research, suggesting that when learners recognize the connection between their present and future selves, they may be more motivated to invest in their future selves and strategically plan their current learning activities, which has been linked to better academic performance (Yang and Zeng, 2022). The mediating role of future self-continuity is also consistent with the career-oriented nature of secondary vocational education. When students perceive a closer connection between their present and future selves, they may be more likely to understand current school learning as relevant to future development, which may support engagement.

### 4.3 The mediating role of academic procrastination

The findings suggest that academic procrastination statistically mediated the association between school climate and learning engagement, thus corroborating H3. Specifically, a positive and supportive school climate was associated with lower academic procrastination and higher learning engagement among secondary vocational students. In situations where students encounter challenging tasks, academic pressure may trigger negative emotions, leading them to adopt procrastination as an immediate strategy for emotion regulation. Within educational settings, positive relationships between teachers and students, as well as among peers, may be associated with students’ tendencies toward academic procrastination (Nordby et al., 2017). One possible explanation is that a positive school environment may be associated with higher psychological capital and academic commitment (Zeng et al., 2022). Under such conditions, students may show greater initiative and punctuality in their learning activities. When faced with difficulties, they are more inclined to seek assistance rather than avoid tasks, thereby reducing their propensity to procrastinate (Zhang et al., 2020).

The current findings indicate a negative correlation between academic procrastination and learning engagement, corroborating previous research (Cerezo et al., 2017; Melgaard et al., 2022). Academic procrastination has been linked to difficulties in completing of learning tasks and to negative emotions, such as anxiety. Prior studies have demonstrated that increased classroom anxiety may disrupt learning processes and impair cognitive functions like attention and memory. It may also be related to lower learning motivation and less effective strategies (Lavasani et al., 2011; Vogel and Schwabe, 2016). Procrastinating students often allocate less time and effort to learning activities, including pre-class preparation, post-class review, and active participation in classroom activities (Jiang, 2024). This pattern may also be understood in relation to students’ need to connect daily school tasks with longer-term educational and occupational goals. When students delay schoolwork, they may have fewer opportunities to experience current learning as purposeful and developmentally relevant. Accordingly, learning support may consider combining study guidance with goal clarification and structured task support.

### 4.4 The chain mediating role of future self-continuity and academic procrastination

The study identified a serial indirect pathway involving future self-continuity and academic procrastination in the association between school climate and learning engagement among secondary vocational students. Specifically, a positive school climate was correlated with enhanced future self-continuity, and future self-continuity was associated with lower academic procrastination and higher learning engagement. These findings corroborate Hypothesis 4 and align with previous research (Blouin-Hudon and Pychyl, 2015; Song et al., 2025), which indicates that individuals with greater future self-continuity tend to report fewer instances of irrational delay.

One possible interpretation is that students with stronger future self-continuity may place greater value on future outcomes and may be less likely to prioritize immediate emotional relief or avoidant coping. In contrast, when individuals perceive a disconnect between their present and future selves, they may prioritize immediate gratification and employ avoidant strategies to evade tasks (Sirois and Pychyl, 2013). Procrastination also reflects a tendency to succumb to immediate mood repair (Tice and Bratslavsky, 2000). When students realize that procrastination may be associated with poorer academic outcomes, they may experience self-blame, helplessness, and frustration (Jia et al., 2021). Such negative emotions may manifest within the classroom setting, potentially diminishing students’ attitudes and engagement (Albulescu et al., 2024).

At the same time, this serial indirect pathway should be interpreted cautiously rather than as a definitive causal mechanism. Alternative explanations are possible: students with higher learning engagement may perceive the school climate more positively, and unmeasured factors such as academic self-efficacy, school belonging, or career clarity may also be related to the observed associations. Thus, the model contributes to existing research by integrating school climate, future self-continuity, and academic procrastination within a serial mediation framework, suggesting that the association between school climate and learning engagement may involve both students’ perceived connection with their future selves and academic procrastination among secondary vocational students.

The serial indirect pathway may therefore be viewed as a tentative pattern linking supportive school environments, future self-continuity, academic procrastination, and engagement in this population. From a practical perspective, secondary vocational schools may consider integrating school climate improvement, career education, and structured learning support to help students connect current learning with future occupational development.

### 4.5 Practical implications

The findings may offer practical implications for secondary vocational schools, educators, and policymakers. The pattern of findings highlights school climate, future self-continuity, and academic procrastination as potential points of attention when developing strategies to support learning engagement in secondary vocational schools. For vocational schools and educators, improving school climate may be considered an important entry point for supporting learning engagement. Schools may strengthen teacher support, peer support, and autonomy opportunities, while teachers may combine emotional support with structured task guidance, such as helping students clarify learning goals, divide tasks into manageable steps, and reflect on the relevance of current learning to future development. Career education may also include illustrative activities, such as guided “dialog with the future self” or growth portfolios based on school learning tasks, to help students connect current learning with future development.

For policymakers and school administrators, the findings suggest that school climate may be considered in vocational education quality improvement, teacher professional development, and student support services. Policies aimed at strengthening career guidance, psychological support, and school belonging may provide supportive conditions for students’ learning engagement. Given the cross-sectional design, these implications should be viewed as practice-oriented suggestions rather than evidence of tested intervention effects.

### 4.6 Limitations and future research directions

The limitations of this study can be delineated into several aspects. Firstly, the study employed a cross-sectional design. Although the serial mediation model was theoretically guided, the cross-sectional design does not permit conclusions about temporal order or causality. Therefore, the mediation results should be interpreted as statistical indirect associations rather than evidence of causal pathways. Future investigations could benefit from adopting longitudinal designs to more effectively explore the dynamic relationships among variables, or by employing cross-lagged panel models to assess the interactions between variables. Secondly, although Harman’s single-factor test did not indicate a dominant common factor, the same-source and same-time self-report design means that potential common method bias should still be acknowledged. Future studies could adopt multi-wave or multi-source designs to further address this limitation. Thirdly, the present study provided additional current-sample evidence for the adopted instruments by reporting CFA fit indices, standardized factor loadings, CR, AVE, and discriminant validity evidence. These results provided support for the psychometric adequacy of the instruments among Chinese secondary vocational students. As this study was not primarily designed as a scale-validation study, future research could continue to examine the construct validity of these instruments in larger and more diverse secondary vocational student samples. Fourthly, the sample characteristics should be considered when interpreting the generalizability of the findings. The participants were recruited from one secondary vocational school in Zhejiang Province through convenience sampling, and the gender composition of the participating program areas contributed to the larger proportion of female students in the sample. Although grade distribution was reported to indicate the upper-secondary vocational education stage of the sample, future studies should collect exact age information to provide a more complete demographic description. Therefore, caution is needed when generalizing the findings to male students, students in other majors, or schools with different program structures. Future studies should adopt multi-school and stratified sampling designs to improve representation across gender groups and vocational majors. Lastly, the present model focused on future self-continuity and academic procrastination as mediators. Future research could extend the model by considering additional explanatory or contextual variables, such as school belonging, academic self-efficacy, career clarity, or teacher autonomy support, and by examining whether these variables function as mediating or moderating factors in different research designs. Future studies could also test the applicability of the model in other secondary vocational schools and neighboring educational contexts, such as general senior high schools and higher vocational colleges.

## 5 Conclusion

This study found that school climate was significantly associated with learning engagement among secondary vocational students in this sample. Future self-continuity and academic procrastination statistically mediated this association, both separately and sequentially. These findings may provide preliminary evidence for potential pathways linking school climate and learning engagement. Given the cross-sectional nature of this study, these findings should be interpreted as associations rather than causal relationships. Furthermore, the findings may offer theoretical and empirical implications for supporting learning engagement in secondary vocational education. Accordingly, secondary vocational schools may consider strengthening school climate and integrating career education with guidance that helps students connect current learning with future development. These practices may be relevant for supporting learning engagement and reducing procrastination-related difficulties, although their intervention effects require further empirical testing.

## Data Availability

The raw data supporting the conclusions of this article will be made available by the authors, without undue reservation.
